# Supplementary material for: Cysteine-Mediated Extracellular Electron Transfer of Lysinibacillus varians GY32
Source: Microbiol Spectr. 2022 Nov 1;10(6):e02798-22. doi: 10.1128/spectrum.02798-22 (PMC9769522; doi:10.1128/spectrum.02798-22)
Supplement: Supplemental file 1 — Supplemental material. Download spectrum.02798-22-s0001.pdf, PDF file, 1.3 MB [file spectrum.02798-22-s0001.pdf]

# Supplementary materials

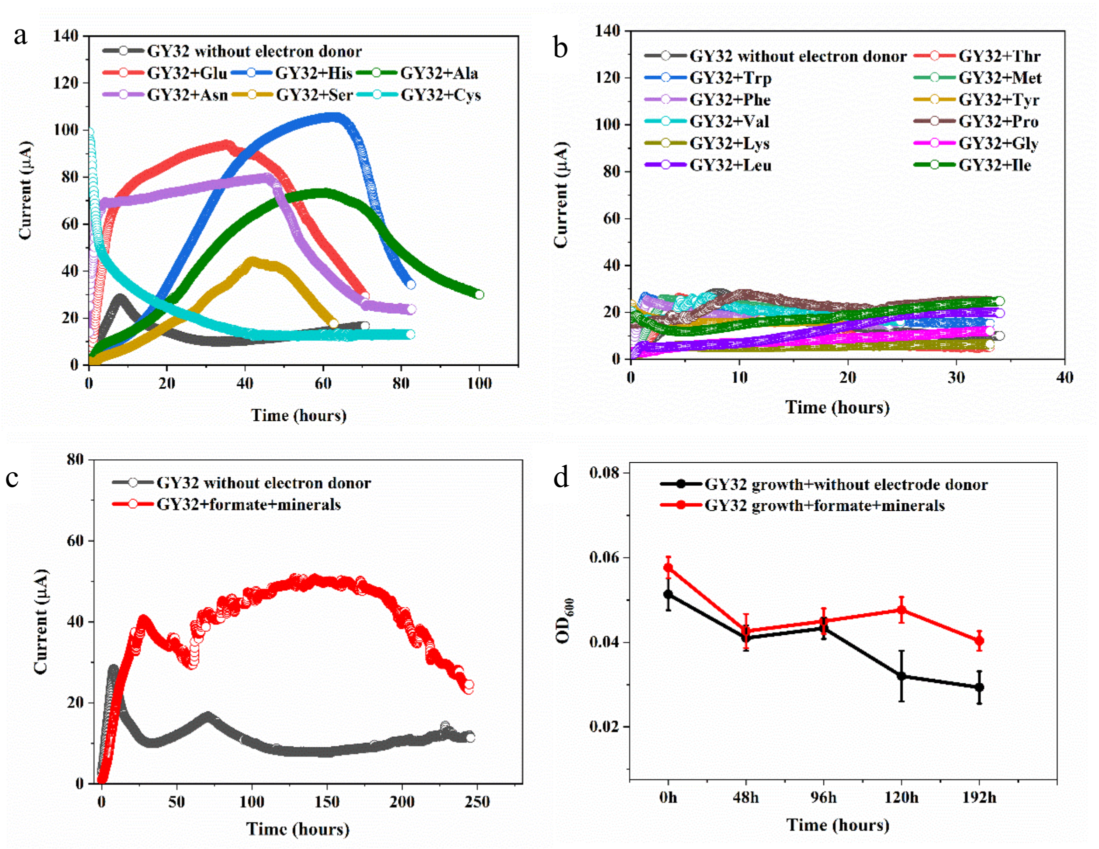


**Fig. S1** The current generation and growth of GY32. (a) The amino acids as electron donors with significant current generation (maximum current > 40 μA). (b) The amino acids as electron donors without significant current generation (maximum current < 30 μA). (c) The current generation of GY32 in a minerals medium with formate as the sole electron donor. (d) The optical density of GY32 in a minerals medium with formate as the sole electron donor.

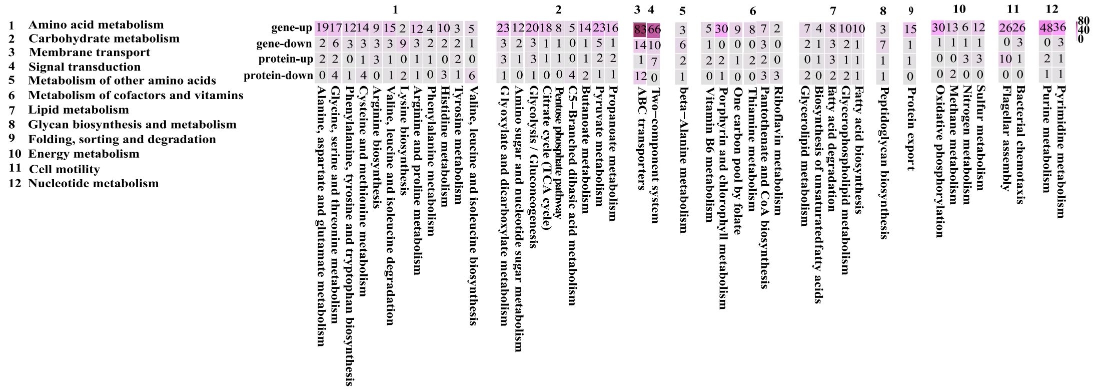


**Fig. S2** The heat map of enriched KEGG pathways of transcriptome and proteomic. The square represents the transcription and expression of significantly different genes enriched in metabolic pathways, and the digits in the square represent the number of genes; the digits at the top of the heat map represent the categories of metabolic pathways.

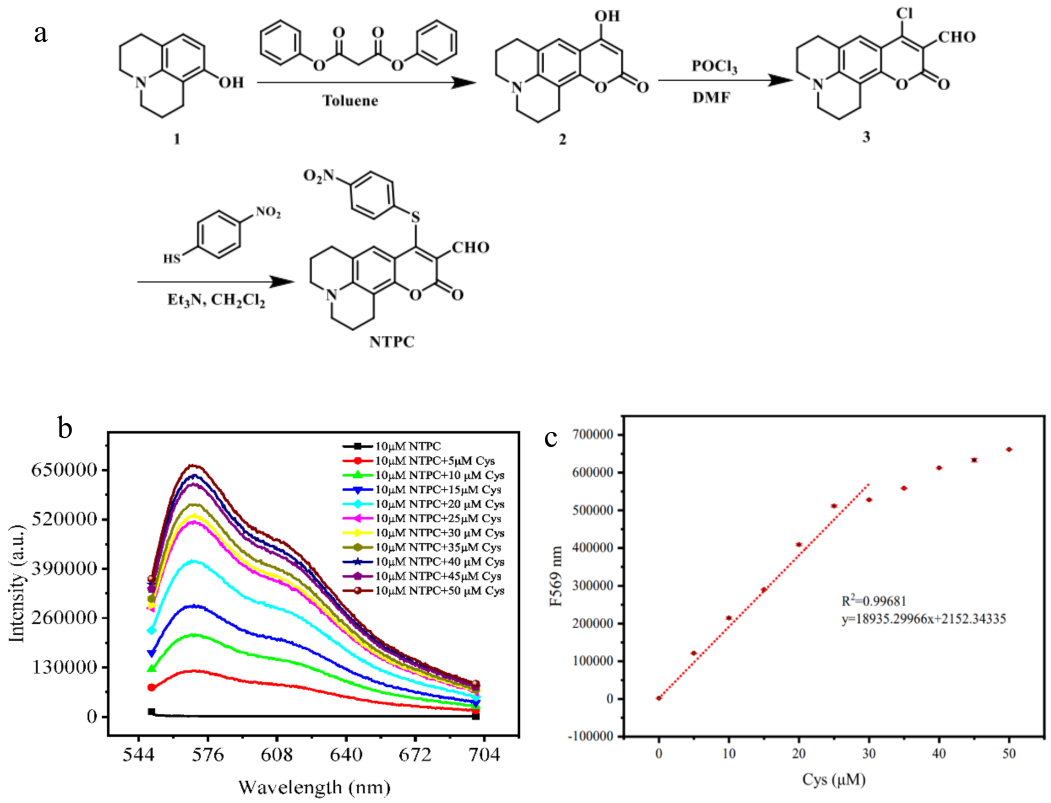


**Fig. S3** The synthesis principle of NTPC and its specificity analysis for cysteine. (a) Brief principle for the synthesis of NTPC. (b) The change of NTPC fluorescence intensity with cysteine concentration. (c) Relationship between NTPC fluorescence intensity and cysteine concentration.

**Table S1** The enrichment pathways in the transcriptome and proteome

| Gene and Pathway | Symbol | Description | Transcriptome [a] | | Proteome [a] | | | |
|---|---|---|---|---|---|---|---|---|
| | | | $\log_2$ (Fold Change) | P-value | $O_2$_mean | MFC_mean | $\log_2$ (Fold Change) | P-value |
| **Oxidative phosphorylation** | | | | | | | | |
| T479_RS16345 | sdhA | succinate dehydrogenase flavoprotein subunit | 3.14 | 1.53E-16 | 33476252 | 36069458.7 | 0.11 | 0.0384 |
| T479_RS16350 | sdhC | succinate dehydrogenase cytochrome B558 | 1.79 | 5.40E-07 | 739432.938 | 945704.021 | 0.35 | 0.2635 |
| T479_RS19870 | cydA | cytochrome D ubiquinol oxidase subunit I | 2.7 | 2.38E-13 | 1020582.1 | 1254551.17 | 0.3 | 0.4579 |
| T479_RS19865 | -- | cytochrome D ubiquinol oxidase subunit II | 3.18 | 3.30E-16 | 3405902.67 | 3591619.17 | 0.08 | 0.0014 |
| T479_RS03775 | ppk | polyphosphate kinase | -1.87 | 7.18E-08 | 14766689 | 16241612.7 | 0.14 | 0.2314 |
| T479_RS02700 | atpB | ATP synthase subunit A | 2.74 | 1.19E-12 | 4923164.75 | 6653501.5 | 0.43 | 0.1073 |
| T479_RS02710 | atpF | F0F1 ATP synthase subunit B | 3.3 | 2.82E-08 | 6069398.17 | 7519160.17 | 0.31 | 0.0937 |
| T479_RS02705 | atpE | ATP synthase F0, C subunit | 3.11 | 2.71E-19 | 243540.922 | 194014.625 | -0.33 | 0.7823 |
| T479_RS02720 | atpA | F0F1 ATP synthase subunit alpha | 4.43 | 4.89E-29 | 65801990.7 | 83585232 | 0.35 | 0 |
| T479_RS02730 | atpD | ATP F0F1 synthase subunit beta | 2.31 | 7.90E-11 | 92778778.7 | 124191603 | 0.42 | 0.0095 |
| T479_RS02715 | atpH | F0F1 ATP synthase subunit delta | 3.23 | 1.65E-12 | 28669422 | 41482168 | 0.53 | 0.211 |
| T479_RS02725 | atpG | F0F1 ATP synthase subunit gamma | 2.04 | 1.86E-08 | 23702786.7 | 30575969.3 | 0.37 | 0.1734 |
| T479_RS04165 | ctaD | cytochrome C oxidase | 2.81 | 1.28E-14 | 20910726.7 | 27141933.3 | 0.38 | 0.1813 |
| T479_RS04160 | ctaC | cytochrome B | 4.21 | 1.19E-24 | 16502686 | 21638351.3 | 0.39 | 0.0083 |
| T479_RS08040 | cbaB | cytochrome B5 | 2.86 | 5.57E-09 | 3662411.92 | 6741045.5 | 0.88 | 0.0191* |
| T479_RS04170 | ctaE | cytochrome B oxidoreductase | 2.25 | 4.28E-09 | 5140402 | 7237759.17 | 0.49 | 0.3361 |
| T479_RS04175 | caaD | cytochrome B6 | 1.49 | 7.41E-04 | 5202845.75 | 6157898.5 | 0.24 | 0.4449 |
| T479_RS14205 | ctaB2 | protoheme IX farnesyltransferase | 4.43 | 2.09E-24 | 140017.695 | 166997.438 | 0.25 | 0.0987 |
| T479_RS15845 | qoxB | quinol oxidase subunit 1 | 4.62 | 1.70E-15 | 2202968.13 | 2511157.67 | 0.19 | 0.4659 |
| T479_RS01205 | yjlD | pyridine nucleotide-disulfide oxidoreductase | 1.62 | 5.51E-06 | 44886738.7 | 50736898.7 | 0.18 | 0.2108 |
| T479_RS17800 | yutJ | NADH dehydrogenase | 5.18 | 3.23E-30 | 2728425.25 | 2807080.83 | 0.04 | 0.5464 |
| T479_RS07295 | qcrA | menaquinol-cytochrome C reductase iron-sulfur subunit | 3.81 | 7.81E-23 | 58057852 | 77608976 | 0.42 | 0 |
| T479_RS07300 | qcrB | cytochrome B6 | 3.65 | 3.95E-20 | 76578437.3 | 98622312 | 0.36 | 0.0001 |
| T479_RS07305 | qcrC | cytochrome Cbb3 | 3.27 | 1.04E-18 | 28296832 | 33076216 | 0.23 | 0.6045 |
| T479_RS20690 | ppaC | inorganic pyrophosphatase | 1.24 | 1.90E-04 | 19834757.3 | 24519453.3 | 0.31 | 0.0096 |
| T479_RS15845 | qoxB | cytochrome aa3-600 menaquinol oxidase subunit I | 4.62 | 1.70E-15 | 2202968.13 | 2511157.67 | 0.19 | 0.465874295 |
| T479_RS15855 | qoxD | cytochrome aa3-600 menaquinol oxidase subunit IV | 2.23 | 5.39E-03 | - | - | - | - |
| T479_RS01205 | ndh | NADH dehydrogenase | 1.62 | 5.51E-06 | 44886738.7 | 50736898.7 | 0.18 | 0.210781741 |
| T479_RS17800 | ndh | NADH dehydrogenase | 5.18 | 3.23E-30 | 2728425.25 | 2807080.83 | 0.04 | 0.546367263 |
| **Riboflavin metabolism** | | | | | | | | |
| T479_RS06350 | ribBA | GTP cyclohydrolase | 1.93 | 9.90E-04 | 1721286.46 | 1637013.54 | -0.07 | 0.95 |
| T479_RS05145 | ribF | riboflavin kinase / FMN adenylyltransferase | 3.56 | 4.75E-13 | 11224379.8 | 18131496.7 | 0.69 | 0.085 |
| T479_RS07705 | ribE | riboflavin synthase subunit alpha | - | - | 1801995.67 | 834620.604 | -1.11 | 1.50E-0** |
| T479_RS07715 | ribH | 6,7-dimethyl-8-ribityllumazine synthase | - | - | 9139315.17 | 3710953.92 | -1.3 | 5.70E-04** |
| T479_RS07710 | ribB | 3,4-dihydroxy-2-butanone-4-phosphate synthase | - | - | 3316403.17 | 1360836.63 | -1.29 | 1.80E-06** |
| **Alanine, aspartate and glutamate metabolism** | | | | | | | | |
| T479_RS16780 | ald | alanine dehydrogenase | 2.12 | 3.44E-10 | 43073114.7 | 43384313.3 | 0.01 | 0.7423 |
| T479_RS08120 | gdh | glutamate dehydrogenase | 3.12 | 8.46E-16 | 3510202.58 | 5354656.33 | 0.61 | 0* |

| | | | | | | | | |
|---|---|---|---|---|---|---|---|---|
| T479_RS04465 | pyrB | aspartate carbamoyltransferase | 1.65 | 1.04E-03 | 3174912.42 | 3547351.08 | 0.16 | 0.2733 |
| T479_RS19960 | purF | amidophosphoribosyltransferase | 4.95 | 2.03E-16 | 2283067.92 | 2115617.13 | -0.11 | 0.2561 |
| T479_RS07845 | aspC | aspartate aminotransferase | 1.26 | 8.01E-04 | 19206822.7 | 21265454.7 | 0.15 | 0.0008 |
| T479_RS21570 | glmS | glucosamine--fructose-6-phosphate aminotransferase | 1.88 | 3.34E-08 | 1681601.96 | 1734431.25 | 0.04 | 0.7675 |
| T479_RS13780 | glsA2 | glutaminase | 2.15 | 6.90E-10 | 484893.76 | 612139.906 | 0.34 | 0.4342 |
| T479_RS11360 | ansB | aspartate ammonia-lyase | 1.2 | 7.96E-04 | 6340189.67 | 6418993.83 | 0.02 | 0.4117 |
| T479_RS00605 | argH2 | argininosuccinate lyase | -1.13 | 3.03E-03 | 960671.083 | 1744160.33 | 0.86 | 0.5531 |
| T479_RS13150 | argH | argininosuccinate lyase | 2.38 | 1.91E-07 | 371391.698 | 308204.771 | -0.27 | 0.5125 |
| T479_RS19985 | purB | adenylosuccinate lyase | 2.24 | 8.70E-08 | 7792354.67 | 8602597.5 | 0.14 | 0.6423 |
| T479_RS19645 | PH0670 | aspartate racemase | 2.14 | 4.31E-09 | 6604407.67 | 8912784 | 0.43 | 0.0675 |
| T479_RS05475 | glnA | glutamine synthetase | 1.97 | 3.65E-06 | 15504521.7 | 17109966.3 | 0.14 | 0.0526 |
| T479_RS06125 | glnA | glutamine synthetase | 1.87 | 2.21E-05 | 1045836.08 | 1709299.83 | 0.71 | 0** |
| T479_RS22535 | purA | adenylosuccinate synthetase | 3.7 | 8.58E-21 | 6796272.5 | 6673352 | -0.03 | 0.1744 |
| T479_RS13155 | argG | argininosuccinate synthase | 2.09 | 6.59E-06 | 1321934.69 | 1293435.71 | -0.03 | 0.9716 |
| T479_RS17065 | asnB | asparagine synthase | 1.71 | 7.25E-07 | 6532280 | 6310015 | -0.05 | 0.0085 |
| T479_RS04480 | carB | carbamoyl phosphate synthase large subunit | 2.27 | 3.15E-08 | 9445946.67 | 8337273.5 | -0.18 | 0 |
| T479_RS04475 | carA | carbamoyl phosphate synthase small subunit | 2.23 | 3.93E-05 | 2632601.25 | 2225612.46 | -0.24 | 0.1414 |
| **Flagellar assembly** | | | | | | | | |
| T479_RS04870 | flgB | flagellar basal body rod protein FlgB | 5.55 | 1.03E-08 | 452387.448 | 543960.031 | 0.27 | 0.0063 |
| T479_RS04875 | flgC | flagellar basal body rod protein FlgC | 4.98 | 9.32E-05 | 174984.122 | 294127.188 | 0.75 | 0** |
| T479_RS04920 | ylxG | flagellar basal body rod modification protein FlgD | 4.55 | 7.54E-05 | 948939.667 | 2590342.17 | 1.45 | 0.0007** |
| T479_RS02770 | flhO | flagellar basal body rod protein FlgG | 1.8 | 4.93E-06 | 621112.115 | 1080376.98 | 0.8 | 0.0021** |
| T479_RS02775 | flhP | flagellar hook-basal body protein | 2.7 | 3.34E-15 | 287269.776 | 887533.563 | 1.63 | 0.0178* |
| T479_RS03120 | flgK | flagellar hook protein FlgK | 3.87 | 9.07E-13 | 1240764.17 | 2163832.17 | 0.8 | 0** |
| T479_RS03125 | flgL | flagellar hook protein FlgL | 2.64 | 5.21E-10 | 1939409.38 | 3014691.33 | 0.64 | 0** |
| T479_RS03110 | -- | anti-sigma factor | 3.43 | 4.34E-04 | 3686829.19 | 4615598.96 | 0.32 | 0.0686 |
| T479_RS04985 | flhA | flagellar biosynthesis protein FlhA | 2.97 | 3.44E-15 | 899875.354 | 1123859.92 | 0.32 | 0.1167 |
| T479_RS03145 | hag | flagellin | 2.41 | 1.25E-10 | 90436368 | 111160344 | 0.3 | 0 |
| T479_RS03205 | fliD | flagellar hook protein | 2.98 | 1.61E-10 | 1277373.67 | 2298850.5 | 0.85 | 0** |
| T479_RS04885 | fliF | flagellar M-ring protein FliF | 2.82 | 1.17E-09 | 2029911.38 | 2898366 | 0.51 | 0 |
| T479_RS04890 | fliG | flagellar motor switch protein FliG | 1.97 | 4.16E-03 | 2625639.67 | 3460690.08 | 0.4 | 0.0929 |
| T479_RS04895 | -- | flagellar assembly protein | 1.84 | 1.08E-02 | 809009.667 | 1148443.94 | 0.51 | 0.585 |
| T479_RS04900 | fliI | MULTISPECIES: ATP synthase | 3.48 | 6.88E-19 | 943945.333 | 1137695.75 | 0.27 | 0.3733 |
| T479_RS04915 | -- | Flagellar hook-length control protein FliK | 14.14 | 3.22E-09 | 898418.646 | 3219909.81 | 1.84 | 0.0004** |
| T479_RS04945 | fliM | flagellar motor switch protein FliM | 5.12 | 2.31E-06 | 1794185.71 | 2040527.83 | 0.19 | 0.0251 |
| T479_RS04960 | fliZ | flagellar protein | 3.02 | 2.37E-08 | 858123.604 | 879665.229 | 0.04 | 0.2779 |
| T479_RS04965 | fliP | flagellar biosynthesis protein FliP | 15.72 | 7.98E-14 | 621070.563 | 787977.208 | 0.34 | 0.0487 |
| T479_RS03210 | fliT | flagellar assembly protein FliT | 2.36 | 2.83E-02 | 491561.24 | 1210282.04 | 1.3 | 0** |
| T479_RS19570 | motA | flagellar motor protein MotA | 4.02 | 8.47E-09 | 3357575.83 | 3754832.25 | 0.16 | 0.691 |
| T479_RS19565 | motB | flagellar motor protein MotB | 3.9 | 3.54E-23 | 2691184.67 | 3239373.58 | 0.27 | 0.0032 |
| T479_RS04995 | ylxH | Flagellar synthesis regulator FleN | 1.33 | 1.13E-03 | 685071.427 | 602846.833 | -0.18 | 0.4367 |
| **Glycine, serine and threonine metabolism** | | | | | | | | |
| T479_RS01875 | hom | homoserine dehydrogenase | -2.07 | 5.09E-10 | 1838834.38 | 1020584.98 | -0.85 | 0.0007** |
| T479_RS18755 | serA | 3-phosphoglycerate dehydrogenase | 3.65 | 9.05E-04 | 5297192.83 | 3143468.75 | -0.75 | 0.0019** |

| | | | | | | | | |
|---|---|---|---|---|---|---|---|---|
| T479_RS13985 | gcvPA | glycine dehydrogenase | 4.52 | 6.79E-18 | 14076664.7 | 20120470 | 0.52 | 0 |
| T479_RS13980 | gcvPB | glycine dehydrogenase subunit 2 | 1.39 | 2.22E-03 | 13504571 | 19669474 | 0.54 | 0 |
| T479_RS06430 | soxA | methyltryptophan oxidase | -1.33 | 2.00E-04 | 425002.104 | 490910.526 | 0.21 | 0.6821 |
| T479_RS04025 | pdhD | dihydrolipoamide dehydrogenase | 2.36 | 4.32E-11 | 44843454.7 | 41287269.3 | -0.12 | 0.0569 |
| T479_RS13525 | bfmBC | dihydrolipoamide dehydrogenase | 2.47 | 3.32E-06 | 14594263 | 16999154.3 | 0.22 | 0.267 |
| T479_RS02675 | glyA | serine hydroxymethyltransferase | 2.12 | 2.79E-08 | 22108466.7 | 32224837.3 | 0.54 | 0.2406 |
| T479_RS13990 | gcvT | glycine cleavage system protein T | 3.58 | 1.24E-15 | 15185627.3 | 29624903.3 | 0.96 | 0** |
| T479_RS14260 | serC | MFS transporter | 2.1 | 7.87E-06 | 12256232.3 | 15624021 | 0.35 | 0.0068 |
| T479_RS21410 | rhbA | 2,4-diaminobutyrate 4-aminotransferase | 3.88 | 4.43E-20 | 1844959.5 | 1020749.9 | -0.85 | 0** |
| T479_RS18815 | glxK | glycerate kinase | 12.54 | 3.00E-04 | 269887.151 | 232378.76 | -0.22 | 0.1614 |
| T479_RS15225 | trpA | tryptophan synthase subunit alpha | -1.23 | 7.25E-04 | 2119496.17 | 3136889.08 | 0.57 | 0.0163 |
| T479_RS02255 | trpB | tryptophan synthase subunit beta | -1.44 | 2.49E-04 | 885773.896 | 984754.5 | 0.15 | 0.0515 |
| T479_RS15230 | trpB | tryptophan synthase subunit beta | -1.85 | 3.52E-06 | 1285166.33 | 1488593.13 | 0.21 | 0.0245 |
| T479_RS20710 | thrC | threonine synthase | -1.19 | 3.65E-04 | 6666528.67 | 5104951.42 | -0.39 | 0.0002 |
| T479_RS04710 | sdaAB | serine dehydratase subunit beta | 1.45 | 1.38E-03 | 884630.667 | 877743.438 | -0.01 | 0.1925 |
| T479_RS02295 | ilvA | threonine dehydratase | 1.73 | 4.36E-06 | 2202459.67 | 1846260.46 | -0.25 | 0.0162 |
| T479_RS02485 | tdcB | threonine dehydratase | 2.62 | 2.16E-04 | 329860.469 | 222283.24 | -0.57 | 0.219 |
| T479_RS18390 | gpmI | phosphoglyceromutase | 3.59 | 3.70E-15 | 4359829.5 | 5230258.17 | 0.26 | 0.5056 |
| T479_RS09600 | gpmB | fructose-2,6-bisphosphatase | 4.5 | 5.95E-18 | 1515546.63 | 954299 | -0.67 | 0.0038 |
| T479_RS12515 | gpmB | phosphoglycerate mutase | 2.35 | 1.40E-04 | 596220.615 | 563453.438 | -0.08 | 0.5998 |
| **Nitrogen metabolism** | | | | | | | | |
| T479_RS08120 | gdh | glutamate dehydrogenase | 3.12 | 8.46E-16 | 3510202.58 | 5354656.33 | 0.61 | 0** |
| T479_RS01435 | SAS0791 | 2-nitropropane dioxygenase | 4.59 | 2.12E-15 | 4408145.58 | 6841223.67 | 0.63 | 0** |
| T479_RS13680 | SH2032 | 2-nitropropane dioxygenase | 2.02 | 3.62E-09 | 6557564.17 | 8080684 | 0.3 | 0.0109 |
| T479_RS05475 | glnA | glutamine synthetase | 1.97 | 3.65E-06 | 15504521.7 | 17109966.3 | 0.14 | 0.0526 |
| T479_RS06125 | glnA | glutamine synthetase | 1.87 | 2.21E-05 | 1045836.08 | 1709299.83 | 0.71 | 0** |
| **Citrate cycle (TCA cycle)** | | | | | | | | |
| T479_RS08020 | mdh | malate dehydrogenase | -1.23 | 1.98E-02 | 2757407 | 3809463.83 | 0.47 | 0.0962 |
| T479_RS16700 | mdh | malate dehydrogenase | 1.03 | 5.65E-03 | 45087006.7 | 46589932 | 0.05 | 0.2195 |
| T479_RS16705 | icd | isocitrate dehydrogenase | 2.82 | 4.64E-15 | 31672364 | 38734846.7 | 0.29 | 0.8307 |
| T479_RS16345 | sdhA | succinate dehydrogenase flavoprotein subunit | 3.14 | 1.53E-16 | 33476252 | 36069458.7 | 0.11 | 0.0384 |
| T479_RS16350 | sdhC | succinate dehydrogenase cytochrome B558 | 1.79 | 5.40E-07 | 739432.938 | 945704.021 | 0.35 | 0.2635 |
| T479_RS16710 | citZ | citrate synthase | 1.83 | 1.96E-07 | 48605510.7 | 43107600 | -0.17 | 0.0011 |
| T479_RS09300 | fumC | fumarate hydratase | 1.6 | 2.58E-06 | 3265273.08 | 2365853.38 | -0.46 | 0.0001 |
| T479_RS09935 | citB | aconitate hydratase | 2.22 | 3.38E-08 | 35690157.3 | 39690550.7 | 0.15 | 0.3056 |
| T479_RS04830 | sucD | succinyl-CoA synthetase subsunit alpha | 2.23 | 4.57E-11 | 49381361.3 | 57301497.3 | 0.21 | 0.1294 |
| T479_RS04825 | sucC | malate--CoA ligase subunit beta | 3.36 | 4.71E-20 | 25332299.3 | 25644659.3 | 0.02 | 0.2625 |
| **ABC transporters** | | | | | | | | |
| T479_RS22140 | braG | amino acid ABC transporter ATPase | 2.84 | 2.14E-10 | 5557175.33 | 2174040.58 | -1.35 | 0** |
| T479_RS22155 | livH | ABC transporter permease | 5.13 | 1.54E-14 | 1737142.42 | 961618.521 | -0.85 | 0.222 |
| T479_RS22150 | braE | ABC transporter | 3.31 | 7.70E-17 | 2286003.88 | 1313396.04 | -0.8 | 0.1579 |
| T479_RS22160 | BruAb1_1767 | ethanolamine utilization protein EutJ | 1.51 | 1.36E-03 | 15916295 | 7540625.25 | -1.08 | 0.1481 |
| T479_RS06340 | opuAA | glycine/betaine ABC transporter ATP-binding protein | 2.29 | 3.31E-03 | 3383604.08 | 4330346.83 | 0.36 | 0.7185 |
| T479_RS06335 | gbuB | MULTISPECIES: glycine/betaine ABC transporter | 2.05 | 7.29E-05 | 6031275.17 | 5976177.67 | -0.01 | 0.9958 |

| | | | | | | | | |
|---|---|---|---|---|---|---|---|---|
| T479_RS06330 | gbuC | MULTISPECIES: glycine/betaine ABC transporter | 1.39 | 8.50E-03 | 4321619.67 | 3422177.08 | -0.34 | 0.0001 |
| T479_RS13305 | cbiN | cobalt transporter CbiN | 3.92 | 2.72E-09 | 504301.18 | 231530.523 | -1.12 | 0.8847 |
| T479_RS00615 | fbpC | spermidine/putrescine ABC transporter ATP-binding protein | -3.22 | 1.10E-14 | 403028.938 | 504840.385 | 0.32 | 0.2126 |
| T479_RS00625 | HI_0131 | iron ABC transporter substrate-binding protein | -2.24 | 9.30E-08 | 494517.875 | 629591.042 | 0.35 | 0.4044 |
| T479_RS01075 | yusV | iron-dicitrate ABC transporter ATP-binding protein | 3.63 | 7.98E-22 | 312065.104 | 401282.755 | 0.36 | 0.2192 |
| T479_RS12530 | cbiZ | iron ABC transporter permease | 2.08 | 1.02E-02 | 403901.646 | 401160.26 | -0.01 | 0.3539 |
| T479_RS16490 | MJ0089 | ABC transporter | 3.78 | 1.18E-21 | 550673.292 | 285443.396 | -0.95 | 0.0036 |
| T479_RS20775 | yclP | iron ABC transporter ATP-binding protein | 3.82 | 7.30E-15 | 2949722.25 | 1763666.46 | -0.74 | 0.816 |
| T479_RS21425 | yusV | iron-enterobactin transporter ATP-binding protein | 2.08 | 1.40E-06 | 184874.151 | 119880.117 | -0.62 | 0.4852 |
| T479_RS16495 | HI_1471 | iron ABC transporter permease | 2.69 | 2.23E-10 | 489122.198 | 97030.1198 | -2.33 | 0.0109* |
| T479_RS20210 | hmuU | ABC transporter permease | 4.04 | 1.31E-10 | 161217.573 | 344721.51 | 1.1 | 0.1107 |
| T479_RS20785 | yclN | iron ABC transporter permease | 4.93 | 5.25E-18 | 693138.313 | 704693.021 | 0.02 | 0.715 |
| T479_RS21435 | yfiZ | siderophore ABC transporter permease | 2.56 | 3.18E-12 | 987305.958 | 212512.01 | -2.22 | 0.0366* |
| T479_RS01090 | fpuA | iron ABC transporter permease | 6.3 | 1.24E-26 | 6651200.33 | 1778185 | -1.9 | 0** |
| T479_RS03565 | isdE | heme ABC transporter substrate-binding protein | 3.04 | 4.93E-13 | 3323927.92 | 3561294.25 | 0.1 | 0.8649 |
| T479_RS20770 | yclQ | iron ABC transporter substrate-binding protein | 3.06 | 2.08E-15 | 30733690.7 | 12067726.7 | -1.35 | 0** |
| T479_RS20935 | yvrC | metal ABC transporter substrate-binding protein | 2.33 | 2.97E-02 | 9718295.67 | 9654038.33 | -0.01 | 0.6709 |
| T479_RS21190 | feuA | iron-uptake system-binding protein | 4.52 | 3.38E-21 | 1298647.38 | 579897.146 | -1.16 | 0.0044** |
| T479_RS21440 | cbrA | iron ABC transporter substrate-binding protein | 2.16 | 3.59E-10 | 7538085.17 | 2153988.58 | -1.81 | 0** |
| T479_RS06770 | modA | molybdenum ABC transporter substrate-binding protein | -1.31 | 5.31E-05 | 1431012.01 | 1498840.48 | 0.07 | 0.7272 |
| T479_RS14110 | pstB | phosphate ABC transporter ATP-binding protein | 1.66 | 6.59E-03 | 26991.5462 | 76864.3542 | 1.51 | 0.0127* |
| T479_RS06315 | pstS1 | membrane protein | 2.61 | 8.67E-06 | 354305 | 500386.719 | 0.5 | 0.0026 |
| T479_RS14125 | pstS | phosphate-binding protein | 3.25 | 2.92E-10 | 275924.615 | 354007.875 | 0.36 | 0.1144 |
| T479_RS17680 | cysA | sulfate ABC transporter | 3.72 | 8.34E-09 | 10040.8776 | 125481.758 | 3.64 | 0.1807 |
| T479_RS17665 | sbp | sulfate transporter subunit | 2 | 1.03E-02 | 406873.953 | 986214.229 | 1.28 | 0.0439* |
| T479_RS02195 | metN2 | methionine ABC transporter ATP-binding protein | 3.42 | 9.99E-07 | 748461.479 | 555027.135 | -0.43 | 0.0078 |
| T479_RS17965 | metN | methionine ABC transporter ATP-binding protein | 2.89 | 1.04E-11 | 3756056.25 | 3773824.5 | 0.01 | 0.0068 |
| T479_RS02205 | metQ | methionine ABC transporter ATPase | 2.05 | 5.36E-04 | 6131074.67 | 3774695.08 | -0.7 | 0* |
| T479_RS17955 | metQ | methionine ABC transporter substrate-binding protein | 1.84 | 3.96E-04 | 5360209.17 | 3083307.33 | -0.8 | 0.0134* |
| T479_RS03275 | ftsX | cell division protein FtsX | 2.64 | 1.12E-06 | 1355991.29 | 1574279.75 | 0.22 | 0.0096 |
| T479_RS17210 | adcA | adhesin | 5.62 | 1.47E-29 | 732085.604 | 1005489.44 | 0.46 | 0.0993 |
| T479_RS14280 | zurA | zinc ABC transporter ATP-binding protein | 4.81 | 5.30E-32 | 2097175.83 | 1897135.58 | -0.14 | 0.9034 |
| T479_RS04565 | oppF | peptide ABC transporter substrate-binding protein | 5.77 | 4.18E-33 | 15118124.7 | 14553294.7 | -0.05 | 0.246 |
| T479_RS03900 | potD | spermidine/putrescine ABC transporter substrate-binding protein | 2.35 | 1.75E-07 | 2427762.25 | 2340642.5 | -0.05 | 0.0854 |
| T479_RS03905 | potC | spermidine/purescine ABC transporter permease | 2.32 | 2.28E-03 | 5487298.83 | 4498591.5 | -0.29 | 0.0428 |
| T479_RS03910 | potB | spermidine/putrescine ABC transporter permease | 2.39 | 7.93E-05 | 1279754.96 | 1139828.27 | -0.17 | 0.3232 |
| T479_RS19460 | msbA | multidrug ABC transporter ATP-binding protein | 3.12 | 4.95E-15 | 366369.125 | 427889.677 | 0.22 | 0.1371 |
| T479_RS12855 | mntA | MULTISPECIES: manganese transporter | -2.88 | 1.32E-15 | 2661451.17 | 1334283.38 | -1 | 0.0891 |
| T479_RS12850 | mntB | manganese ABC transporter ATP-binding protein | -3.33 | 2.91E-14 | 3505580.42 | 2070921.33 | -0.76 | 0** |
| T479_RS04585 | oppA | ABC transporter substrate-binding protein | 3.82 | 4.93E-21 | 43384362.7 | 41351761.3 | -0.07 | 0.1436 |

| | | | | | | | | |
|---|---|---|---|---|---|---|---|---|
| T479_RS08980 | aliB | peptide ABC transporter substrate-binding protein | -2.16 | 6.16E-03 | 81664.1471 | 351189.729 | 2.1 | 0.1896 |
| T479_RS04580 | oppB | peptide ABC transporter permease | 5.46 | 1.74E-34 | 11452647.3 | 11416381.3 | 0 | 0.2896 |
| T479_RS04575 | oppC | diguanylate cyclase | 6.66 | 2.47E-37 | 6288203.83 | 5679089.5 | -0.15 | 0.0638 |
| T479_RS04570 | oppD | peptide ABC transporter ATP-binding protein | 5.38 | 3.88E-32 | 11014870.7 | 9857582.67 | -0.16 | 0.2249 |
| T479_RS10350 | nikA | sodium:proton antiporter | 2.06 | 1.51E-03 | 843646.042 | 790870.417 | -0.09 | 0.7088 |
| T479_RS12920 | nikA | nickel ABC transporter substrate-binding protein | 3.39 | 1.13E-02 | 148672.984 | 180412.412 | 0.28 | 0.6093 |
| T479_RS21745 | ecfT | cobalt ABC transporter permease | 3.27 | 9.51E-09 | 574562.229 | 467227.094 | -0.3 | 0.3279 |
| T479_RS21755 | ecfA | cobalt transporter ATP-binding subunit | 3.15 | 1.54E-11 | 1683909.71 | 1742363.79 | 0.05 | 0.6493 |
| T479_RS21750 | ecfA2 | cobalt transporter ATP-binding subunit | 5.09 | 3.35E-10 | 1047727.75 | 1217996.23 | 0.22 | 0.0998 |
| T479_RS20835 | ytrE | ABC transporter ATP-binding protein | 2.73 | 3.19E-06 | 436249.146 | 734762.74 | 0.75 | 0.2395 |
| T479_RS01130 | tcyP | L-cystine transport | - | - | 897230.438 | 781700.021 | -0.19 | 0.0103 |
| T479_RS16540 | tcyK | L-cystine-binding protein | - | - | 232167.401 | 86695.3776 | -1.42 | 0.0198** |
| T479_RS21245 | expZ | elongation factor 3 | -1.18 | 5.00E-03 | 597668.927 | 673780.021 | 0.17 | 0.1566 |
| **beta-Alanine metabolism** | | | | | | | | |
| T479_RS01945 | aldHT | aldehyde dehydrogenase | -2.58 | 1.24E-10 | 3190713 | 4549634.83 | 0.51 | 0.0231 |
| T479_RS03655 | ALDH3A2 | aldehyde dehydrogenase | 1.24 | 1.69E-04 | 6593787.17 | 8080740 | 0.29 | 0.1801 |
| T479_RS03800 | iolA2 | methylmalonate-semialdehyde dehydrogenase | -1.49 | 6.02E-03 | 2154893.83 | 2913517 | 0.44 | 0.0039 |
| T479_RS09630 | Acad10 | acyl-CoA dehydrogenase | 4.27 | 1.46E-22 | 437387.948 | 514535.896 | 0.23 | 0.0783 |
| T479_RS07420 | hyuA | phenylhydantoinase | -2.13 | 4.12E-03 | 122545.396 | 353411.708 | 1.53 | 0.0004** |
| T479_RS07380 | panC | pantoate--beta-alanine ligase | - | - | 132305.162 | 439437.391 | 1.73 | 0.0034** |
| **Arginine biosynthesis** | | | | | | | | |
| T479_RS08120 | gdh | glutamate dehydrogenase | 3.12 | 8.46E-16 | 3510202.58 | 5354656.33 | 0.61 | 0** |
| T479_RS16565 | argF | ornithine carbamoyltransferase | 2.8 | 9.10E-13 | 597819.792 | 813485.333 | 0.44 | 0.012 |
| T479_RS07845 | aspC | aspartate aminotransferase | 1.26 | 8.01E-04 | 19206822.7 | 21265454.7 | 0.15 | 0.0008 |
| T479_RS13780 | glsA2 | glutaminase | 2.15 | 6.90E-10 | 484893.76 | 612139.906 | 0.34 | 0.4342 |
| T479_RS21605 | rocF | arginase | 14.15 | 6.59E-08 | 2179119.75 | 3400667 | 0.64 | 0.0018** |
| T479_RS00605 | argH2 | argininosuccinate lyase | -1.13 | 3.03E-03 | 960671.083 | 1744160.33 | 0.86 | 0.5531 |
| T479_RS13150 | argH | argininosuccinate lyase | 2.38 | 1.91E-07 | 371391.698 | 308204.771 | -0.27 | 0.5125 |
| T479_RS05475 | glnA | glutamine synthetase | 1.97 | 3.65E-06 | 15504521.7 | 17109966.3 | 0.14 | 0.0526 |
| T479_RS06125 | glnA | glutamine synthetase | 1.87 | 2.21E-05 | 1045836.08 | 1709299.83 | 0.71 | 0** |
| T479_RS13155 | argG | argininosuccinate synthase | 2.09 | 6.59E-06 | 1321934.69 | 1293435.71 | -0.03 | 0.9716 |
| **Valine, leucine and isoleucine degradation** | | | | | | | | |
| T479_RS03745 | ykwC | oxidoreductase | 2.18 | 2.31E-10 | 6368538.83 | 6224004.5 | -0.03 | 0.2384 |
| T479_RS01945 | aldHT | aldehyde dehydrogenase | -2.58 | 1.24E-10 | 3190713 | 4549634.83 | 0.51 | 0.0231 |
| T479_RS03655 | ALDH3A2 | aldehyde dehydrogenase | 1.24 | 1.69E-04 | 6593787.17 | 8080740 | 0.29 | 0.1801 |
| T479_RS03800 | iolA2 | methylmalonate-semialdehyde dehydrogenase | -1.49 | 6.02E-03 | 2154893.83 | 2913517 | 0.44 | 0.0039 |
| T479_RS13515 | bfmBAB | 2-oxoisovalerate dehydrogenase subunit beta | 1.55 | 2.22E-06 | 34298898.7 | 34810372 | 0.02 | 0.964 |
| T479_RS09630 | Acadm | acyl-CoA dehydrogenase | 4.27 | 1.46E-22 | 437387.948 | 514535.896 | 0.23 | 0.0783 |
| T479_RS13530 | ldh | leucine dehydrogenase | 4.06 | 1.72E-25 | 39335904 | 43711268 | 0.15 | 0.3944 |
| T479_RS04025 | pdhD | dihydrolipoamide dehydrogenase | 2.36 | 4.32E-11 | 44843454.7 | 41287269.3 | -0.12 | 0.0569 |
| T479_RS13525 | bfmBC | dihydrolipoamide dehydrogenase | 2.47 | 3.32E-06 | 14594263 | 16999154.3 | 0.22 | 0.267 |
| T479_RS02545 | mmgA | acetyl-CoA acetyltransferase | 6.21 | 2.53E-45 | 15750143.3 | 15852836.7 | 0.01 | 0.5183 |
| T479_RS17995 | fadA | acetyl-CoA acetyltransferase | 3.87 | 1.44E-20 | 2930885.67 | 3903342.08 | 0.41 | 0.4829 |
| T479_RS02155 | hmgL | hydroxymethylglutaryl-CoA lyase | 3.91 | 3.82E-08 | 1509281.21 | 1584232.17 | 0.07 | 0.5879 |
| T479_RS13500 | mutB | methylmalonyl-CoA mutase | 3.79 | 3.63E-15 | 18085954 | 16452386.3 | -0.14 | 0.0553 |

| | | | | | | | | |
|---|---|---|---|---|---|---|---|---|
| T479_RS13505 | mutA | methylmalonyl-CoA mutase | 3.13 | 3.05E-11 | 15402042 | 13145886 | -0.23 | 0.0003 |
| T479_RS13480 | PH0272 | lactoylglutathione lyase | 2.21 | 2.47E-08 | 7438652 | 7547006.17 | 0.02 | 0.9004 |
| T479_RS02570 | icmF | methylmalonyl-CoA mutase | 4.82 | 1.88E-32 | 21447015.3 | 17147371.3 | -0.32 | 0 |
| **Arginine and proline metabolism** | | | | | | | | |
| T479_RS01945 | aldHT | aldehyde dehydrogenase | -2.58 | 1.24E-10 | 3190713 | 4549634.83 | 0.51 | 0.0231 |
| T479_RS03655 | ALDH3A2 | aldehyde dehydrogenase | 1.24 | 1.69E-04 | 6593787.17 | 8080740 | 0.29 | 0.1801 |
| T479_RS08590 | proA | gamma-glutamyl phosphate reductase | 2.01 | 3.82E-04 | 2517560.25 | 3446355.25 | 0.45 | 0.0033 |
| T479_RS07845 | aspC | aspartate aminotransferase | 1.26 | 8.01E-04 | 19206822.7 | 21265454.7 | 0.15 | 0.0008 |
| T479_RS17545 | rocD | ornithine-oxoacid aminotransferase | 2.33 | 3.36E-06 | 14513469.3 | 21566564 | 0.57 | 0 |
| T479_RS08595 | proB | gamma-glutamyl kinase | 12.4 | 1.07E-03 | 1653043.5 | 1515373.71 | -0.13 | 0.0358 |
| T479_RS10995 | pip | proline iminopeptidase | 4.1 | 6.63E-04 | 1461942.79 | 939764.646 | -0.64 | 0.0031** |
| T479_RS08445 | nylA | amidase | -1.82 | 3.13E-06 | 279427.396 | 312492.563 | 0.16 | 0.6299 |
| T479_RS21605 | rocF | arginase | 14.15 | 6.59E-08 | 2179119.75 | 3400667 | 0.64 | 0.0018 |
| T479_RS02430 | speB | agmatinase | 3.76 | 1.77E-21 | 4635590.17 | 4937725 | 0.09 | 0.767 |
| **Bacterial chemotaxis** | | | | | | | | |
| T479_RS07245 | cheR | chemotaxis protein CheR | 2.75 | 9.13E-07 | 1039674.63 | 1233403.96 | 0.25 | 0.3763 |
| T479_RS22325 | cheR | chemotaxis protein R | 2.06 | 2.21E-04 | 121562.037 | 263350.271 | 1.12 | 0.3512 |
| T479_RS04890 | fliG | flagellar motor switch protein FliG | 1.97 | 4.16E-03 | 2625639.67 | 3460690.08 | 0.4 | 0.0929 |
| T479_RS04945 | fliM | flagellar motor switch protein FliM | 5.12 | 2.31E-06 | 1794185.71 | 2040527.83 | 0.19 | 0.0251 |
| T479_RS19570 | motA | flagellar motor protein MotA | 4.02 | 8.47E-09 | 3357575.83 | 3754832.25 | 0.16 | 0.691 |
| T479_RS19565 | motB | flagellar motor protein MotB | 3.9 | 3.54E-23 | 2691184.67 | 3239373.58 | 0.27 | 0.0032 |
| T479_RS00775 | tlpC | chemotaxis protein | 1.47 | 1.39E-05 | 1078778.46 | 1660039.79 | 0.62 | 0.0011** |
| T479_RS00865 | pctA | chemotaxis protein | 3.03 | 5.52E-09 | 7710417.67 | 7460426.17 | -0.05 | 0.9093 |
| T479_RS00880 | BT9727_0355 | methyl-accepting chemotaxis protein | -1.49 | 3.70E-03 | 55165.4284 | 88305.4063 | 0.68 | 0.0548 |
| T479_RS06870 | tlpC | methyl-accepting chemotaxis protein | 1.56 | 6.70E-03 | 4592763.17 | 5447379 | 0.25 | 0.0044 |
| T479_RS07500 | hemAT | chemotaxis protein | 2.18 | 2.34E-02 | 3617806.83 | 3530260.83 | -0.04 | 0.2044 |
| T479_RS07580 | mcpA | chemotaxis protein | 4.4 | 1.65E-04 | 531780.531 | 497400.052 | -0.1 | 0.5838 |
| T479_RS09065 | mcpA | MULTISPECIES: chemotaxis protein | 2.72 | 3.27E-10 | 2483821.67 | 3692201.58 | 0.57 | 0 |
| T479_RS11250 | bdlA | YoaH | -1.82 | 3.12E-05 | 578444.448 | 321906.745 | -0.85 | 0.1247 |
| T479_RS11355 | -- | methyl-accepting chemotaxis protein | 3.47 | 1.78E-13 | 5646399.17 | 5986375.33 | 0.08 | 0.279 |
| T479_RS19750 | bdlA | chemotaxis protein | 2.25 | 1.30E-03 | 512571.375 | 589767.188 | 0.2 | 0.3904 |
| T479_RS19795 | mcpA | methyl-accepting chemotaxis protein | 3.72 | 6.55E-03 | 1375198.92 | 1235161.96 | -0.15 | 0.1057 |
| T479_RS20295 | BT9727_0355 | methyl-accepting chemotaxis protein | 1.46 | 2.55E-03 | 5785413.5 | 6132436.5 | 0.08 | 0.2117 |
| T479_RS20895 | tlpC | chemotaxis protein | 4.18 | 8.43E-11 | 695167.833 | 675175.188 | -0.04 | 0.433 |
| T479_RS21070 | mcpA | methyl-accepting chemotaxis protein | 1.11 | 2.07E-03 | 4813117 | 3307508.67 | -0.54 | 0 |
| T479_RS22285 | mcpB | chemotaxis protein | 2.74 | 5.91E-06 | 1190763.04 | 1390660.21 | 0.22 | 0.321 |
| T479_RS22895 | -- | chemotaxis protein | 12.12 | 1.39E-02 | 637393.125 | 434405.656 | -0.55 | 0.0073 |
| T479_RS05005 | cheA | chemotaxis protein CheA | 3.25 | 3.91E-12 | 8004500.5 | 9410588.33 | 0.23 | 0.863 |
| T479_RS22330 | luxQ | histidine kinase | 4.4 | 3.30E-09 | 2202963 | 2237903.71 | 0.02 | 0.3462 |
| T479_RS05015 | cheC | chemotaxis protein CheY | 2.42 | 1.78E-09 | 4061695.25 | 3268805.67 | -0.31 | 0.0267 |
| T479_RS05020 | cheD | chemotaxis protein CheD | 2.48 | 1.03E-06 | 1138235.71 | 1226666.92 | 0.11 | 0.1409 |
| T479_RS04955 | cheY | chemotaxis protein cheY-like protein | 2.78 | 2.96E-13 | 4717018.67 | 5157548.83 | 0.13 | 0.3312 |
| T479_RS08780 | cheY | chemotaxis protein CheY | 2.46 | 3.59E-12 | 1257356.96 | 1639014.29 | 0.38 | 0.018 |
| **Histidine metabolism** | | | | | | | | |
| T479_RS01945 | aldHT | aldehyde dehydrogenase | -2.58 | 1.24E-10 | 3190713 | 4549634.83 | 0.51 | 0.0231 |

| | | | | | | | | |
|---|---|---|---|---|---|---|---|---|
| T479_RS03655 | ALDH3A2 | aldehyde dehydrogenase | 1.24 | 1.69E-04 | 6593787.17 | 8080740 | 0.29 | 0.1801 |
| T479_RS00910 | hutI | imidazolonepropionase | 3.57 | 5.50E-15 | 7534136.33 | 4767445.67 | -0.66 | 0** |
| T479_RS09620 | hutG | formimidoylglutamase | 3.67 | 4.78E-10 | 1843076.71 | 657553.083 | -1.49 | 0** |
| T479_RS00905 | hutU | urocanate hydratase | 2.72 | 4.20E-14 | 17801216 | 14406423.7 | -0.31 | 0 |
| T479_RS00895 | hutH | histidine ammonia-lyase | 3.74 | 1.05E-22 | 16472390 | 8190441 | -1.01 | 0** |
| T479_RS15045 | HISN3 | phosphoribosylformimino-5-aminoimidazole carboxamide ribotide isomerase | 1.7 | 8.20E-05 | 2360279.79 | 3466911.83 | 0.55 | 0.1132 |
| T479_RS18505 | hisF | imidazole glycerol phosphate synthase | 2.04 | 1.83E-06 | 1263097.38 | 1016380.15 | -0.31 | 0.297 |
| T479_RS18500 | hisI | phosphoribosyl-ATP pyrophosphatase | 3.12 | 1.32E-03 | 5790784.17 | 5962826.67 | 0.04 | 0.3119 |
| **Pyruvate metabolism** | | | | | | | | |
| T479_RS08105 | ytsJ | NAD-dependent malic enzyme 4 | 1.34 | 2.24E-04 | 6514989 | 5824461.58 | -0.16 | 0.2431 |
| T479_RS16485 | Ldhd | 2-hydroxy-acid oxidase | -1.19 | 6.87E-04 | 2781510.75 | 2417031.92 | -0.2 | 0.8082 |
| T479_RS19825 | Ldhd | MULTISPECIES: 2-hydroxy-acid oxidase | -1.12 | 6.16E-03 | 1839612.17 | 1492097.83 | -0.3 | 0.0467 |
| T479_RS01945 | aldHT | aldehyde dehydrogenase | -2.58 | 1.24E-10 | 3190713 | 4549634.83 | 0.51 | 0.0231 |
| T479_RS03655 | ALDH3A2 | aldehyde dehydrogenase | 1.24 | 1.69E-04 | 6593787.17 | 8080740 | 0.29 | 0.1801 |
| T479_RS04010 | pdhA | pyruvate dehydrogenase E1 subunit alpha | 4.4 | 1.49E-24 | 30443836.7 | 41382388 | 0.44 | 0 |
| T479_RS04015 | pdhB | 2-oxoisovalerate dehydrogenase subunit beta | 2.76 | 9.13E-13 | 36473602.7 | 49826341.3 | 0.45 | 0.0233 |
| T479_RS04025 | pdhD | dihydrolipoamide dehydrogenase | 2.36 | 4.32E-11 | 44843454.7 | 41287269.3 | -0.12 | 0.0569 |
| T479_RS13525 | bfmBC | dihydrolipoamide dehydrogenase | 2.47 | 3.32E-06 | 14594263 | 16999154.3 | 0.22 | 0.267 |
| T479_RS02545 | mmgA | acetyl-CoA acetyltransferase | 6.21 | 2.53E-45 | 15750143.3 | 15852836.7 | 0.01 | 0.5183 |
| T479_RS04020 | pdhC | branched-chain alpha-keto acid dehydrogenase subunit E2 | 4.32 | 4.04E-24 | 38300300 | 50582037.3 | 0.4 | 0.001 |
| T479_RS16725 | pyk | pyruvate kinase | 1.46 | 5.61E-05 | 20818788.7 | 17917838.7 | -0.22 | 0.0124 |
| T479_RS10835 | -- | acylphosphatase | 3.32 | 1.56E-03 | 2779730.67 | 4645026.42 | 0.74 | 0.0103* |
| T479_RS17175 | pckA | phosphoenolpyruvate carboxykinase | 1.12 | 9.78E-04 | 34561846 | 44274612 | 0.36 | 0 |
| T479_RS19540 | -- | MULTISPECIES: fumarate hydratase | 1.43 | 9.80E-06 | 22089276 | 27421314.7 | 0.31 | 0.8422 |
| T479_RS22280 | yetH | glyoxalase | 2.09 | 9.39E-05 | 4353618.17 | 5161018.33 | 0.25 | 0.2605 |
| T479_RS16810 | ytcI | acyl--CoA ligase | 3.56 | 3.90E-18 | 3546286.92 | 3254396.33 | -0.12 | 0.605 |
| T479_RS21640 | ytcI | acyl--CoA ligase | 1.47 | 2.55E-03 | 9052214.83 | 6148904.25 | -0.56 | 0.0034 |
| T479_RS04145 | pyc | pyruvate carboxylase | 1.85 | 3.44E-08 | 12917601.7 | 9561489.67 | -0.43 | 0 |
| T479_RS02145 | accC | biotin carboxylase | 1.68 | 2.16E-05 | 11861353.3 | 9499003.67 | -0.32 | 0 |
| T479_RS13645 | accC1 | acetyl-CoA carboxylase biotin carboxylase subunit | 2.41 | 1.93E-12 | 45495825.3 | 41127277.3 | -0.15 | 0.228 |
| T479_RS13650 | accB | acetyl-CoA carboxylase | 1.85 | 4.73E-04 | 31621023.3 | 33911134.7 | 0.1 | 0.6957 |
| T479_RS20585 | cat1 | acetyl-CoA hydrolase | 2.2 | 4.68E-08 | 10583288.7 | 7562924.17 | -0.48 | 0 |
| **Pantothenate and CoA biosynthesis** | | | | | | | | |
| T479_RS04295 | panE | 2-dehydropantoate 2-reductase | 2.9 | 8.69E-07 | 341204.214 | 415948.271 | 0.29 | 0.6147 |
| T479_RS16645 | coaE | dephospho-CoA kinase | 1.67 | 3.70E-03 | 3995593.88 | 4068814.75 | 0.03 | 0.8106 |
| T479_RS04255 | coaD | phosphopantetheine adenylyltransferase | 2.28 | 9.83E-05 | 1587334.25 | 1614118.54 | 0.02 | 0.2498 |
| T479_RS07420 | hyuA | phenylhydantoinase | -2.13 | 4.12E-03 | 122545.396 | 353411.708 | 1.53** | 0.0004** |
| T479_RS13070 | ilvB | acetolactate synthase catalytic subunit | 1.8 | 1.52E-04 | 4268948.42 | 2727847.58 | -0.65 | 0** |
| T479_RS00455 | coaX | pantothenate kinase | 2 | 6.65E-05 | 477350.469 | 445555.979 | -0.1 | 0.6156 |
| T479_RS05755 | sfp | 4-phosphopantetheinyl transferase | 3.54 | 2.59E-07 | 1637888.25 | 1906357.92 | 0.22 | 0.1448 |
| T479_RS08175 | coaW | pantothenate kinase | 1.55 | 2.62E-04 | 2319985.08 | 3054477 | 0.4 | 0.9899 |
| **Valine, leucine and isoleucine biosynthesis** | | | | | | | | |
| T479_RS13050 | leuB | 3-isopropylmalate dehydrogenase | 2.4 | 4.43E-05 | 3705449.92 | 1553031.88 | -1.25 | 0** |

| | | | | | | | | |
|---|---|---|---|---|---|---|---|---|
| T479_RS13530 | ldh | leucine dehydrogenase | 4.06 | 1.72E-25 | 39335904 | 43711268 | 0.15 | 0.3944 |
| T479_RS13070 | ilvB | acetolactate synthase catalytic subunit | 1.8 | 1.52E-04 | 4268948.42 | 2727847.58 | -0.65 | 0** |
| T479_RS13045 | leuC | isopropylmalate isomerase | 2.1 | 2.32E-05 | 1965268.29 | 1130393.44 | -0.8 | 0** |
| T479_RS02295 | ilvA | threonine dehydratase | 1.73 | 4.36E-06 | 2202459.67 | 1846260.46 | -0.26 | 0.0162 |
| T479_RS02485 | tdcB | threonine dehydratase | 2.62 | 2.16E-04 | 329860.469 | 222283.24 | -0.57 | 0.219 |
| **Sulfur and Cysteine metabolism** | | | | | | | | |
| T479_RS17685 | cysI | sulfite reductase | 2.9 | 9.52E-13 | 456613.177 | 1002331.77 | 1.13 | 0** |
| T479_RS00470 | cysK | cysteine synthase | - | - | 39481210.7 | 28365602.7 | -0.48 | 0 |
| T479_RS17645 | cysC | adenylylsulfate kinase | 4.06 | 1.86E-16 | 444895.604 | 806239.432 | 0.86 | 0.0111** |
| T479_RS17650 | sat | sulfate adenylyltransferase | 3.08 | 4.16E-10 | 81737.6875 | 484467.649 | 2.57 | 0.03* |
| T479_RS17660 | cysH | phosphoadenosine phosphosulfate reductase | 2.48 | 3.84E-07 | | | - | - |
| T479_RS22025 | cysE | serine acetyltransferase | - | - | 1928416.75 | 2990046.83 | 0.63 | 0.007** |
| T479_RS03930 | ykuV | thiol-disulfide oxidoreductase | 2.24 | 2.84E-08 | 2051792.92 | 3325674.58 | 0.70 | 0.0091** |
| ***C*-type cytochrome** | | | | | | | | |
| T479_RS14495 | -- | cytochrome C551 | 3.03 | 1.56E-18 | 2456153.75 | 3413293.25 | 0.48 | 0.5139 |
| T479_RS07305 | -- | cytochrome Cbb3 | 3.26 | 1.04E-18 | 28296832 | 33076216 | 0.23 | 0.6045 |
| T479_RS04160 | -- | cytochrome B | 4.21 | 1.19E-24 | 16502686 | 21638351.3 | 0.39 | 0.0083 |
| T479_RS18555 | -- | CccB | 1.46 | 6.95E-05 | 6018906 | 8976130.17 | 0.58 | 0.9709 |

[a] Results are generated by comparing electrode grown cells with oxygen grown cells. Proteins expression (peptide signal intensity) are presented as the mean value (“$O_2$_mean” and “MFC_ mean”). For “$\log_2$ (Fold Change)”, the dash (–) means undetected. Positive and negative values indicate up- and down-regulated expression in MFC, respectively. * P-value ≤ 0.05 & $|\log_2$ (Fold Change)$|$ ≥ 0.58, ** P-value ≤ 0.01& $|\log_2$ (Fold Change)$|$≥ 0.58.

**Table S2** The CXXCH motif in *c*-type cytochrome

| Symbol | Description | CXXCH motif | Position | Amino acid number |
|---|---|---|---|---|
| T479_RS06590 | cytochrome C | 1 | Inner membrane | 442 |
| T479_RS20980 | cytochrome c | 2 | Inner membrane /Periplasm | 316 |
| T479_RS18555 | CccB | 1 | membrane | 108 |
| T479_RS07305 | cytochrome Cbb3 | 1 | Inner membrane | 257 |
| T479_RS04160 | cytochrome c oxidase subunit II | 1 | Inner membrane | 362 |
| T479_RS14495 | cytochrome C551 | 1 | membrane | 116 |
